# Distribution of superantigens in group A streptococcal isolates from Salvador, Brazil

**DOI:** 10.1186/1471-2334-14-294

**Published:** 2014-05-29

**Authors:** Hillary F Berman, Sara Yee Tartof, Joice N Reis, Mitermayer G Reis, Lee W Riley

**Affiliations:** 1 Division of Infectious Disease and Vaccinology, School of Public Health, University of California, Berkeley, CA 94720, USA; 2 Kaiser Permanente Southern California Research and Evaluation, Pasadena, CA 91101, USA; 3 Gonçalo Moniz Research Center, Oswaldo Cruz Foundation, Salvador, Brazil

**Keywords:** *Streptococcus pyogenes*, Streptococcal superantigens, Group A streptococcus, *Emm* types

## Abstract

**Background:**

Group A streptococcus (GAS) causes invasive disease, superficial disease, and can asymptomatically colonize humans. Superantigens are one virulence factor found in GAS. Previous studies found associations between the genes that encode superantigens and *emm* type of GAS. It is unknown if these associations are due to underlying biological factors that limit the distribution of superantigens or, alternatively, if these associations are due to the expansion of local GAS linages where these studies took place. To further address this question we screened GAS isolates collected from Salvador, Brazil for 11 known superantigen genes.

**Methods:**

Seventy-seven GAS isolates were screened by PCR for superantigen genes. These superantigen genes were *speA*, *speC*, *speG*, *speH*, *speI*, *speJ*, *speK*, *speL*, *speM*, *ssa*, and *smeZ*. We used Fisher’s two-sided exact test to identify associations between superantigens and GAS *emm* type. We then compared our results to previous reports of superantigen prevalence and superantigen association with *emm* type.

**Results:**

In our collection we found several *emm* type and superantigen genotype combinations that have previously been reported in isolates from Europe and Australia. We also found that *speA* was significantly associated with *emm* type 1, and that *speC* was significantly associated with *emm* type 12.

**Conclusions:**

Our study reports superantigen genotypes of GAS from a region of the world that is lacking this information. We found evidence of common GAS superantigen genotypes that are spread worldwide as well as novel superantigen genotypes that, so far, are unique to Brazil.

## Background

*Streptococcus pyogenes* (Group A Streptococcus, or GAS) is a Gram positive bacterium that causes a wide spectrum of clinical manifestations including pharyngitis, skin infections, necrotizing fasciitis, and streptococcal toxic shock syndrome [1]. GAS harbors many virulence factors including superantigens [1].

Superantigens are bacterial exotoxins that are able to activate large numbers of T cells [1-3]. There are eleven known superantigens found in *S. pyogenes*: SpeA, SpeC, SpeG, SpeH, SpeI, SpeJ, SpeK, SpeL, SpeM, SSA, and SmeZ [4]. Superantigen genotype varies by *emm* type and the region of the world in which the isolate was collected [5,6]. An area of active research is whether these associations are due to factors that limit the distribution of superantigens or are due to the expansion of local GAS linages [7]. To further address this question we studied a collection of GAS isolates from Brazil. Previous studies of superantigens in GAS isolates from this region of the world are limited and none of these studies have reported the genotypes of all eleven superantigens [8].

Some superantigens are encoded by genes located in the prophage region of the GAS genome. Others are encoded by genes located outside of this region and are considered to be chromosomally encoded [7,9,10]. The *SpeA*, *speC*, *SpeH*, *speI*, *speK, speL, speM* and *ssa* genes have been found within prophages in streptococcal isolates [4,7,11]. The *speG*, *SpeJ*, and *smeZ* genes are considered to be encoded chromosomally [9,12].

The aim of this project was to determine if associations between superantigens, superantigen genotypes, and *emm* types that have been reported elsewhere were also found in our collection of Group A *streptococcus* isolates collected from Salvador, Brazil. These isolates were collected as part of a previous study of Group A Streptococcus in children with and without pharyngitis [13,14]. From this previously published study we selected all isolates of the two most common *emm* types (*emm* 1 and *emm* 12) and the *emm* type with the strongest association with pharyngitis (*emm* 66) to genotype [13].

## Methods

### Isolate collection and emm typing

*S. pyogenes* isolates were collected and *emm* typing was conducted as part of a separate cross-sectional study detailed elsewhere [13,14]. Briefly, isolates were collected from three hospital clinics in Salvador, Brazil. Patients between the ages of three and fifteen were recruited from the middle of April 2007 to end of October 2008 [13,14]. As described in previous publications, Institutional Review Board (IRB) approval was obtained from all hospitals, the Comissão Nacional de Ética em Pesquisa (Conep) (National Bioethics Commission of Brazil), the Comitê de Ética em Pesquisa-Centro de Pesquisa Gonçalo Moniz-Fiocruz (Ethics Committee for Research - Fiocruz), and the University of California, Berkeley Committee for the Protection of Human Subjects [13,14]. As previously described consent was obtained from parents or guardians and verbal consent was obtained from children [13,14]. Isolates were collected from children who had pharyngitis (strep throat) and those who were asymptomatic carriers of GAS. *Emm* typing of all isolates was performed as described by the Center for Disease Control and Prevention (CDC) protocol http://www.cdc.gov/ncidod/biotech/strep/protocol_emm-type.htm.

We selected the two most common *emm* types (*emm* 1; n = 25 and *emm* 12; n = 40) as well as the *emm* type with strongest association with pharyngitis (*emm* 66; n = 12) to analyze for superantigen genes. For the purposes of this analysis the *emm* 1.25 isolate (n = 1) was included with *emm* 1.

### Superantigen identification by PCR

Superantigen genes were identified by PCR using primers previously published by Maripuu *et al.* or Commons *et al.*[6,15] (Table 1). The Commons *et al.* primers were used to identify *speB* (a positive control for DNA quality), *speJ*, *speK*, *speH*, *speL*, and *speM*. The Maripuu *et al.* primers were used to identify *speA*, *speC*, *speG*, *speI*, *ssa*, and *smeZ*. Each 20 μl PCR mixture contained 1 U of taq DNA polymerase (New England Biolabs, Ipswich, MA), 0.25 mM DNTPs (New England Biolabs, Ipswich, MA), 1X ThermoPol buffer (New England Biolabs, Ipswich, MA), 1 μl template DNA, and 1 nanomole of the forward and reverse primer. Template DNA was extracted with the Qiagen DNEasy kit as previously described [13]. The cycling program for the Commons *et al.* primers was as follows: 2 minutes at 95°C, 35 cycles of 30 seconds at 94°C, 30 seconds at 50°C, 60 seconds at 72°C, and a final extension for 2 minutes at 72°C. The cycling program used for Maripuu *et al.* was as follows: 5 minutes at 94°C, 25 cycles of 30 seconds at 94°C, 30 seconds at primer specific annealing temperature, 60 seconds at 72°C, and a final extension of 7 minutes at 72°C. As a positive control for each superantigen gene PCR, we used the DNA of an isolate known to contain the relevant superantigen gene. We were unable to amplify *speL* from any isolate and therefore were unable to generate a positive control for *speL*.

**Table 1:** Primers used

**Gene**	**Forward primer (5′ to 3′)**	**Reverse primer (5′ to 3′)**	**Annealing temp. °C**	**Product size (bp)**
*speA*1-3,6 [15]	5′-GGACTAACAATCTCGCAAGAGG-3′	5′-TTACTTGGTTGTTAGGTAGACTTC-3′	54	696
*speA*4*,*5 [15]	5′-GCTAACAACCTCACAAGAAG -3′	5′-TGCTTGAGTCAAGCGTCTC-3′	53	659
*speC*[15]	5′-GATTTCTACTATTTCACC-3′	5′-AAATATCTGATCTAGTCCC-3′	44	584
*speG*[15]	5′-CTTATGCAGATGAAAATTTAAAAGATT-3′	5′-AAAGCAAAGGGGGGAGAATAG-3′	53.2	664
*speI*[15]	5′-ATGAGTAGTGTGGGAGTTATTAA-3′	5′-ATGAAGTTGATCAGAATAAGCG-3′	55	645
*speJ*[15]	5′-ATCTTATTTAGTCCAAAGGTAAAT-3′	5′-GTGAACGAGGAGAGGTATGAA-3′	55.5	718
*smeZ*[15]	5′-TTGGATTAGAAGTAGATAA-3′	5′-TTGAATTAGGAGTCAATTTCTATATCT-3′	52	638
*ssa*[15]	5′-AGTCAGCCTGACCCTACTCCA-3′	5′-TAAGGTGAACCTCTATAGCTATAG-3′	59.1	691
*speH*[6]	5′-TTGGATCCAATTCTTATAATACAACC-3′	5′-CCACTTCCTGAGCGGTTACTTTCGG-3′	50	399
*speJ*[6]	5′-CTATGGTGGAATTACACC-3′	5′-CATGTTTATTGCCATTGATCGC-3′	50	247
*speK*[6]	5′-GCGGATCCGATACGTACAATACAAATG-3′	5′-GCGAATTCAATAGCATTCAACCA-3′	50	798
*speL*[6]	5′-GCAGCCATATGGAAGAGACTATTCCTCTTAAGGATATATACTCTC-3′	5′-GGGGATCCTTAATTTTCTTTGTTTGTGAATAAATAGAC-3′	50	703
*speM*[6]	5′-GCAGCCATATGTTTTCAGATGCTGTGTTGG-3′	5′-GGGGATCCTTAATTTTTAGAAAAATCTTCGTTTAAGTA-3′	50	661
*speB*[6]	5′-GGATCCCAACCAGTTGTTAAATCTCT-3′	5′-AACGTTCTAAGGTTTGATGCCTACAA-3′	50	774

All primer sequences shown here have previously been published by Maripuu *et al.* or Commons *et al.*

### Literature review

To the best of our knowledge, we included all studies in our literature review that tested for the presence or absence of the GAS superantigen genes that showed variability in our study. These superantigens are *speA*, *speC*, *speI*, *speJ*, *speM*, and *ssa*. We excluded any studies in which superantigen genotypes of individual isolates could not be determined.

### Data analysis

The associations between superantigens and categorical variables were analyzed by Fisher’s two-sided exact test. STATA (StataCorp, Version 10) was used for all analyses.

## Results and discussion

### Distribution of superantigen genes

We identified *speA, speC, speG, speH, speI, speJ, speM, ssa*, and *smeZ* gene sequences from at least one isolate. We did not find any isolates with *speK* or *speL* in the collection (Table 2). The most common gene, *speG*, was present in all isolates. The *smeZ* gene was present in 99% of 77 isolates. The least common superantigen genes that were identified in at least one strain were *speM*, found in 5% isolates, and *ssa*, found in 8% isolates.

**Table 2:** **Distribution of superantigens by *emm *type**

	**Total (N = 77)**	**emm1 (N = 25)**	**emm12 (N = 40)**	**emm66 (N = 12)**	**P-value**
*speA*	32 (42%)	25 (100%)	1 (3%)	6 (50%)	p < .001
*speC*	33 (43%)	0	33 (83%)	0	p < .001
*speG*	77 (100%)	25 (100%)	40 (100%)	12 (100%)	
*speH*	28 (36%)	0	22 (55%)	6 (50%)	p < .001
*speI*	48 (62%)	4 (16%)	37 (93%)	7 (58%)	p < .001
*speJ*	11 (14%)	9 (36%)	1 (3%)	1 (8%)	p < .001
*speK*	0	0	0	0	
*speL*	0	0	0	0	
*speM*	4 (5%)	1 (4%)	3 (8%)	0	
*ssa*	6 (8%)	1 (4%)	4 (10%)	1 (8%)	
*smeZ*	76 (99%)	25 (100%)	40 (100%)	11 (92%)	

The *speA* genes in *emm* 1 strains were identified by the Maripuu *et al. speA* 1*–*3, 6 primers. The *speA* genes in *emm* 12 and *emm* 66 strains were identified by the Maripuu *et al. speA*-4, 5 primers, indicating they were different alleles. The prevalence of superantigen genes varied across *emm* type (Table 2). The *speA* gene was found in 100% of *emm* 1 isolates, 50% of *emm* 66 isolates, and in only 3% of *emm* 12 isolates (p < .001). *SpeC* was found in 83% of *emm* 12 isolates and in no *emm* 1 or *emm* 66 isolates (p < .001). The *speH* gene was found in 55% of *emm* 12 isolates, 50% of *emm* 66 isolates, and in no *emm* 1 isolates (p < .001). The *speI* gene was found in 93% of *emm* 12 isolates, 58% of *emm* 66 isolates, and in 16% of *emm* 1 isolates (p < .001). The *speJ* gene was found in 3% of *emm* 12 isolates, 8% of *emm* 66 isolates, and in 36% of *emm* 1 isolates (p < .001).

### Superantigen-emm genotyping

We examined the diversity of superantigen genotypes within the same *emm* type. We were able to distinguish 20 distinct strains of GAS using superantigen genotypes alone (Table 3). The three *emm* types we examined in this study were further divided into 24 distinct genotypes by the combination of *emm* typing and superantigen genotype. We reviewed previously reported *emm* 1, 12, and 66 superantigen genotypes and found many genotypes have been given multiple names. For further reference, we have included the original citation and superantigen genotype name from all studies included in our literature review (Table 4). For the subsequent analysis all the genotypes names used are from Table 3.

**Table 3:** **Superantigen genotype and distribution across *emm *type**

**Genotype**	***speA***	***speC***	***speG***	***speH***	***speI***	***speJ***	***speK***	***speL***	***speM***	***ssa***	***smeZ***	**emm12 (40)**	**emm1 (25)**	**emm66 (12)**	**p-value**
A (n = 1)	X		X		X				X		X	0	1 (4%)	0	
B (n = 17)	X		X								X	0	13 (52%)	4 (33.3%)	<.001
C (n = 1)	X		X			X				X	X	0	1 (4%)	0	
D (n = 7)	X		X			X					X	0	7 (28%)	0	<.001
E (n = 3)	X		X		X						X	0	2 (8%)	1 (8.3%)	
F (n = 1)	X		X		X	X					X	0	1 (4%)	0	
G (n = 1)			X	X	X				X		X	1 (2.5%)	0	0	
H (n = 2)		X	X	X	X					X	X	2 (5%)	0	0	
J (n = 19)		X	X	X	X						X	19 (47.5%)	0	0	<.001
K (n = 3)		X	X								X	3 (7.5%)	0	0	
M (n = 2)		X	X	X	X				X		X	2 (5%)	0	0	
N (n = 8)			X	X	X						X	5 (12.5%)	0	3 (25%)	
O (n = 1)	X	X	X	X	X						X	1 (2.5%)	0	0	
P (n = 1)			X								X	0	0	1 (8.3%)	
Q (n = 4)		X	X		X						X	4 (10%)	0	0	
R (n = 1)		X	X	X	X	X					X	1 (2.5%)	0	0	
S (n = 1)		X	X		X					X	X	1 (2.5%)	0	0	
T (n = 1)			X	X	X	X						0	0	1 (8.3%)	
U (n = 2)			X	X	X					X	X	1 (2.5%)	0	1 (8.3%)	
V (n = 1)	X		X	X	X						X	0	0	1 (8.3%)	

Superantigen genotypes B and D were found significantly more often in *emm* type 1 isolates. Superantigen genotype J was found significantly more often in *emm* type 12 isolates. P-values were generated with the two-sided Fisher’s exact test.

**Table 4:** Previously reported superantigen genotypes

**Genotype**	**speA**	**speC**	**speG**	**speH**	**speI**	**speJ**	**speK**	**speL**	**speM**	**ssa**	**smeZ**	**Othername**	**Countries**	**emm**	**Year**
A (n = 1)	X		X		X				X		X	None			
B (n = 17)	X		X								X	R	Australia	1	2001-2002 [6]
												A	Sweden	1	1989 [15]
C (n = 1)	X		X			X				X	X	F	Australia	1	2001-2001 [6]
D (n = 7)	X		X			X					X	D	Australia	1	2001-2002 [6]
												C	Sweden	1	1988-2001 [15]
												Not Given	Norway	1	2006-2007 [16]
												Not Given	New Zealand	1	Not given [17]
												Not Given	Spain	1	1999-2003 [18]
												I	Norway	1	1988-2003 [19]
												Not Given	Sweden	1	2006-2007 [20]
E (n = 3)	X		X		X						X	None			
F (n = 1)	X		X		X	X					X	None			
G (n = 1)			X	X	X				X		X	None			
H (n = 2)		X	X	X	X					X	X	D	China	1	2005-2006 [21]
												D	China	12	1993-1994, 2005-2006 [21]
J (n = 19)		X	X	X	X						X	H	Australia	12	2001-2002 [6]
												S3	Sweden	12	1989-1993 [15]
												Not Given	Norway	12	2006-2007 [16]
												Not Given	Spain	12	1999-2003 [18]
												VII	Norway	12	1988-2003 [19]
												S26	Sweden	66	1989 [15]
K (n = 3)		X	X								X	None			
M (n = 2)		X	X	X	X				X		X	None			
N (n = 8)			X	X	X						X	J	Australia	12	2001-2002 [6]
												Not Given	Norway	12	2006-2007 [16]
												Not Given	Spain	12	1999-2003 [18]
												AC	Sweden	66	1988-1990 [15]
O (n = 1)	X	X	X	X	X						X	None			
P (n = 1)			X								X	None			
Q (n = 4)		X	X		X						X	None			
R (n = 1)		X	X	X	X	X					X	Not Given	ATCC	1	Not given [17]
S (n = 1)		X	X		X					X	X	None			
T (n = 1)			X	X	X	X						Not Given	New Zealand	12	Not given [17]
U (n = 2)			X	X	X					X	X	T	Australia	12	2001-2002 [6]
												Not Given	Spain	12	1999-2003 [18]
V (n = 1)	X		X	X	X						X	None			

Previously published *emm* 1, 12 and 66 superantigen genotypes were reviewed and compared to our collection. “Genotype” refers to designations given in this paper. “Other name” refers to pattern name given in previously published work. Citations refer to the reference section.

The most common superantigen genotype, J, contained 19 isolates. The second most common genotype, B, contained 17 isolates. Genotype J was exclusively found in *emm* 12, and represented 48% of all *emm* 12 isolates (p < .001). Genotype B was significantly associated with *emm* 1 (p < .001) but was also found in 4 *emm* 66 isolates. Genotype D was exclusively found in *emm* 1 isolates (p < .001).

### Discussion

In this study *speA* and *speJ* were significantly associated with *emm* 1. *speH* and *speI* were significantly less likely to be found in *emm* 1 isolates as compared to the rest of the collection (Table 2). Previous studies also found that *speA* is frequently found in *emm* type 1 GAS isolates [6,15,22,23]. We found that superantigen genotype B and superantigen genotype D comprised the majority of *emm* 1 isolates. Genotype B and genotype D differ only by the presence of *speJ* in genotype D. Genotype B has previously been reported in *emm* 1 isolates from Australia between 1989 and 2002 [6]. Genotype D has previously been reported exclusively in *emm* 1 isolates. These isolates were collected in Norway, Spain, New Zealand, and Australia between 1988 and 2003 [6,15,16,18,19].

In this study *speC* was significantly associated with *emm* 12 and was not found in either *emm* 1 or e*mm* 66 isolates. Previous studies found *speC* is often found in *emm* 12 isolates including isolates from China and Japan [23,24]. However, in contrast to the results of the current study, the studies of isolates from China and Japan have found that *speC* is also present in the *emm*1 isolates from these same collections [23,24].

In the current study, the majority of *emm* 12 isolates had superantigen genotype J. Genotype J has previously been reported in *emm* 12 and 66 from Norway, Spain, and Australia between 1989 and 2007, indicating that this particular genotype is very common and has spread worldwide [6,15,16,18,19]. There were no statistically significant associations of superantigens with *emm* 66. Only 12 *emm* 66 isolates were included in this study and the sample size may have been too small to produce meaningful results.

## Conclusions

Previous studies found superantigens are not randomly distributed across *emm* types [6,15,22]. However, associations between specific superantigens and *emm* types often vary by study. Previous studies have suggested numerous hypotheses to explain this variation. It is unknown if this variation is due to underlying biological factors that limit the distribution of superantigens, the selective advantage due to carrying particular superantigens, or the chance expansion of local GAS clones at the time these studies took place. This is an area of active research [7,11]. The current study helps to address this question by reporting the superantigen genotypes of GAS isolated from a region of the world from which information on superantigen genotypes is lacking. This is the first study to report the prevalence of all 11 superantigens in a collection of isolates from South America or Brazil. Similar to other previously published work we found some common genotypes of GAS which are spread world wide as well as novel genotypes of GAS which have only been reported in this study.

## Competing interests

The authors declare that they have no competing interests.

## Authors’ contributions

HB and ST carried out the lab work and statistical analysis required for the study. HB drafted the manuscript. LW, JR, and MR provided critical review of the manuscript and participated in the study designed. All authors read and approved the final manuscript.

## Pre-publication history

The pre-publication history for this paper can be accessed here:

http://www.biomedcentral.com/1471-2334/14/294/prepub
